# Screening and Diagnosis of Malnutrition in Individuals With Obesity: A Scoping Review of Current Methods

**DOI:** 10.1111/obr.70033

**Published:** 2025-11-19

**Authors:** Natasha Nalucha Mwala, Jos W. Borkent, Carliene van Dronkelaar, Jeanne J. F. A. in 't Hulst, Barbara S. van der Meij, Maarten R. Soeters, Marian A. E. de van der Schueren

**Affiliations:** ^1^ Department of Nutrition, Dietetics and Lifestyle, School of Allied Health HAN University of Applied Sciences Nijmegen The Netherlands; ^2^ Division of Human Nutrition and Health Wageningen University and Research Wageningen The Netherlands; ^3^ Department of Endocrinology and Metabolism, Internal Medicine Amsterdam University Centers Amsterdam The Netherlands; ^4^ Nutrition and Dietetics Research Group Bond University Gold Coast Australia

**Keywords:** diagnostic methods, malnutrition screening, nutritional assessment, obesity, undernutrition

## Abstract

**Rationale:**

The global rise in obesity presents a major public health challenge, commonly associated with an increased risk of noncommunicable diseases. Paradoxically, individuals with obesity, particularly older adults and those with comorbidities, are also at risk of malnutrition. This coexistence, driven by inadequate nutritional intake, chronic inflammation, and immune dysfunction, highlights the need to understand these overlapping health risks. Obesity complicates the identification and management of malnutrition. This review examines current screening and diagnostic methods for malnutrition in individuals with obesity.

**Methods:**

A systematic scoping review was conducted following the Joanna Briggs Institute guidelines and Preferred Reporting Items for Systematic Reviews and Meta‐Analyses extension for Scoping Reviews. Literature was searched using a comprehensive strategy across the EBSCOhost database.

**Results:**

From 2097 search results, 41 studies with 420,498 participants met the inclusion criteria. Three main methods for assessing malnutrition risk/nutritional status emerged: blood markers, malnutrition screening tools, and physical/etiologic assessments. The diagnostic criteria described were typically based on healthy weight reference values, lacking obesity‐specific cutoff values. Only two studies introduced tools tailored to individuals with obesity: the Nutrition Health Outcomes Questionnaire and the Just a Nutritional Screening Tool.

**Conclusion:**

Current malnutrition screening and diagnostic methods lack reliability, validity, and appropriate reference values for individuals with obesity. This limits their effectiveness in accurately identifying malnutrition risk in this population. Adjusting cutoff values for key indicators such as weight loss and muscle mass is vital to improve the accuracy of malnutrition diagnosis and ensure appropriate clinical management for individuals with obesity.

**Abbreviations**
BIA: bioelectrical impedance analysis
BMI: body mass index
CONUT: controlling nutritional status
COVID‐19: Coronavirus disease 2019
CPI: calf proportion index
CRP: C‐reactive protein
CT: computed tomography
DEXA: dual‐energy X‐ray absorptiometry
ESPEN: European Society for Clinical Nutrition and Metabolism
GLIM: Global Leadership Initiative on Malnutrition
HGS: hand grip strength
ICD‐10‐AM: International Classification of Diseases, 10th Revision, Australian Modification
IL‐6: interleukin‐6
JaNuS: just a nutrition screening
JBI: Joanna Briggs Institute
MNA: mini nutritional assessment
MNA‐SF: mini nutritional assessment—short form
MST: malnutrition screening tool
MUAC: middle upper arm circumference
MUST: malnutrition universal screening tool
NAFLD: nonalcoholic fatty liver disease
NHOQ: nutrition health outcomes questionnaire
NRS 2002: nutritional risk screening 2002
NUTRIC: nutrition risk in the critically ill
PEM: protein‐energy malnutrition
PG‐SGA: patient‐generated subjective global assessment
PG‐SGA‐SF: patient‐generated subjective global assessment—short form
PNI: prognostic nutritional index
PRISMA‐ScR: Preferred Reporting Items for Systematic Reviews and Meta‐Analyses extension for Scoping Reviews
SGA: subjective global assessment
WHO: World Health Organization

## 1 Introduction

The global prevalence of overweight and obesity (hereafter referred to as “obesity”) continues to rise, presenting a major public health challenge [1, 2]. Obesity is commonly associated with conditions such as metabolic syndrome and, subsequently, cardiovascular disease and type 2 diabetes [3]. However, obesity is progressively being recognized as a factor that complicates malnutrition risk screening, diagnosis, and management [4]. In this paper, malnutrition refers specifically to undernutrition, including protein‐energy malnutrition (PEM).

Recent events, including the COVID‐19 pandemic, have highlighted the heightened vulnerability of individuals with obesity, who experienced more adverse clinical outcomes, including higher rates of severe illness, hospitalization, and mortality [5]. This vulnerability is further magnified when obesity coexists with malnutrition, which has been associated with poorer clinical outcomes such as impaired immune response, prolonged hospital stays, and increased mortality [4]. Reduced or inadequate nutritional intake, chronic inflammation, and immune dysfunction, all exacerbated by the COVID‐19 pandemic, contribute to the development of malnutrition in individuals with obesity [6]. This highlights the need for a comprehensive understanding of the health risks associated with obesity, including the paradoxical coexistence of obesity and malnutrition.

COVID‐19 further revealed how systemic inflammation in individuals with obesity increases disease severity [5, 6]. Obesity compromises immune function, increases the risk of various diseases, and places great strain on the cardiovascular and respiratory systems [7, 8]. Moreover, altered pharmacokinetics and heightened susceptibility to side effects from treatments such as chemotherapy or immunotherapy may reduce therapeutic effectiveness [9, 10]. Consequently, managing chronic conditions in individuals with obesity becomes more complex, leading to higher surgical risks and accelerated progression of diseases such as congestive heart failure and diabetes [11].

Obesity and malnutrition often coexist [12, 13], presenting a paradox that complicates the screening and diagnosis of affected individuals. Malnutrition is usually overlooked in individuals with obesity because body mass index (BMI) is typically relied upon as the primary risk factor [14, 15]. As most malnutrition screening tools focus on low BMI [16], individuals with a high BMI may be misclassified or underdiagnosed. Additionally, weight loss criteria used in malnutrition assessments may not be appropriate for individuals with obesity, as they often require a larger absolute weight loss to meet percentage‐based thresholds, potentially delaying or missing the identification of clinically significant weight loss [4, 17, 18]. Assessment is further complicated in these individuals by their greater muscle mass, which makes detecting muscle mass loss using standard absolute cutoff values challenging [19, 20]. As a result, malnutrition in individuals with obesity frequently goes unrecognized [21].

PEM, characterized by an inadequate or imbalanced intake of energy and protein, remains a global health concern as it is the most common form of disease‐related malnutrition [22, 23]. It is associated with a range of adverse health outcomes, including increased hospitalization rates, compromised immune function, and higher mortality rates [24, 25]. Effective malnutrition screening is essential for identifying individuals at risk of PEM and guiding subsequent diagnostic assessments [14]. According to the Global Leadership Initiative on Malnutrition (GLIM) criteria, these assessments should follow a positive risk screening to confirm malnutrition and determine its severity [26]. However, current screening tools often face challenges with false‐negative outcomes, particularly in individuals with obesity, where traditional indicators of malnutrition do not apply [21].

To address these challenges, we conducted a scoping review to examine the screening and diagnostic methods used to identify malnutrition risk and diagnose malnutrition in individuals with obesity. This review critically assesses the applicability of these methods, considering their strengths and limitations within the context of obesity.

## 2 Research Questions

The scoping review aimed to address two key research questions:

- What screening methods have been used to identify malnutrition risk in individuals with obesity?
- How has malnutrition in individuals with obesity been diagnosed?

## 3 Methods

This review adhered to the Joanna Briggs Institute (JBI) guidelines for scoping reviews, specifically following the framework outlined by Peters et al. [27]. Additionally, it followed the Preferred Reporting Items for Systematic Reviews and Meta‐Analyses extension for Scoping Reviews (PRISMA‐ScR) checklist [28]. Certain checklist items of the PRISMA‐ScR intended for meta‐analyses were omitted, as they were beyond the scope of this review. No quality or risk of bias assessment was conducted because these were not part of our research objectives and are not mandatory for a scoping review [29]. As scoping reviews are typically iterative, we chose not to publish a protocol in advance [30].

### 3.1 Data Collection

A comprehensive search was performed on EBSCOhost, a widely used academic research database, to identify articles published up until 2024 that aligned with the review's objectives and eligibility criteria. Thereafter, an information specialist assisted in developing a search string for Medline, specifically designed to address the scoping review's research questions. A proximity operator (n5) was applied to limit results to studies where these terms appear within five words of each other. This was done not only because “malnutrition” and “weight loss” are common keywords in nutrition research but also because relevant studies on malnutrition in obesity are more likely to use these words near each other. This strategy focused on identifying studies that specifically addressed malnutrition in the context of obesity. The search string included the following terms: “obes* n5 maln*) OR (obes* n5 undern*) OR (overweigh* n5 maln*) OR (overweigh* n5 undern*) OR (overfeed* n5 maln*) OR (overfeed* n5 undern*) OR (obes* n5 nutrition* screening) OR (overweigh* n5 nutrition* screening) OR (overfeed* n5 nutrition* screening) OR (obes* n5 nutrition* assessment) OR (overweigh* n5 nutrition* assessment) OR (overfeed* n5 nutrition* assessment).”

The search results were exported to EndNote [31] before being imported into Rayyan [32], a systematic review web‐based tool designed to assist with the screening and selection of studies. The study selection process followed the JBI guidelines and involved two independent researchers (NNM and JWB), who determined the eligibility of studies using a two‐step process. First, relevant articles were screened based on their titles and abstracts. Then, studies meeting the inclusion and exclusion criteria were selected (Table 1). Next, full texts were reviewed to confirm eligibility. Disagreements were resolved through discussion and consensus by NNM and JWB.

**TABLE 1:** Inclusion and exclusion criteria for studies on malnutrition screening and diagnosis in individuals with obesity.

Inclusion criteria	Exclusion criteria
• Adults aged ≥ 18 years with obesity (BMI ≥ 25 kg/m^2^)	• Studies involving pregnant women
• A clear definition of malnutrition in the methods section, including specific diagnostic criteria or cutoff values (excluding general statements without defined thresholds)	• Studies focusing solely on micronutrient malnutrition
• Terms “overweight,” “obesity” or “bariatric surgery” in the title (added during the iterative process)	• Studies published in languages other than English
• Results stratified by BMI (added during the iterative process)	• Secondary sources (e.g., reviews)
	• Case reports
	• Abstract‐only publications

### 3.2 Data Extraction

NNM and JWB independently extracted relevant information from the included studies using a standardized data extraction form. The extracted data were then thoroughly cross‐checked to ensure the accuracy of all essential details. Any disagreements were resolved through discussion, with MdvdS consulted when necessary.

The following data were extracted: Article characteristics (first author, year of publication, and country of study), population and sample size, care setting, age, method(s) or tool(s) used to screen for and diagnose malnutrition, and any relevant remarks regarding the validity and applicability of these method(s) or tool(s) to identify malnutrition in a population with obesity. This information was then synthesized into a summary table.

## 4 Results

### 4.1 Search Results

**FIGURE 1:**
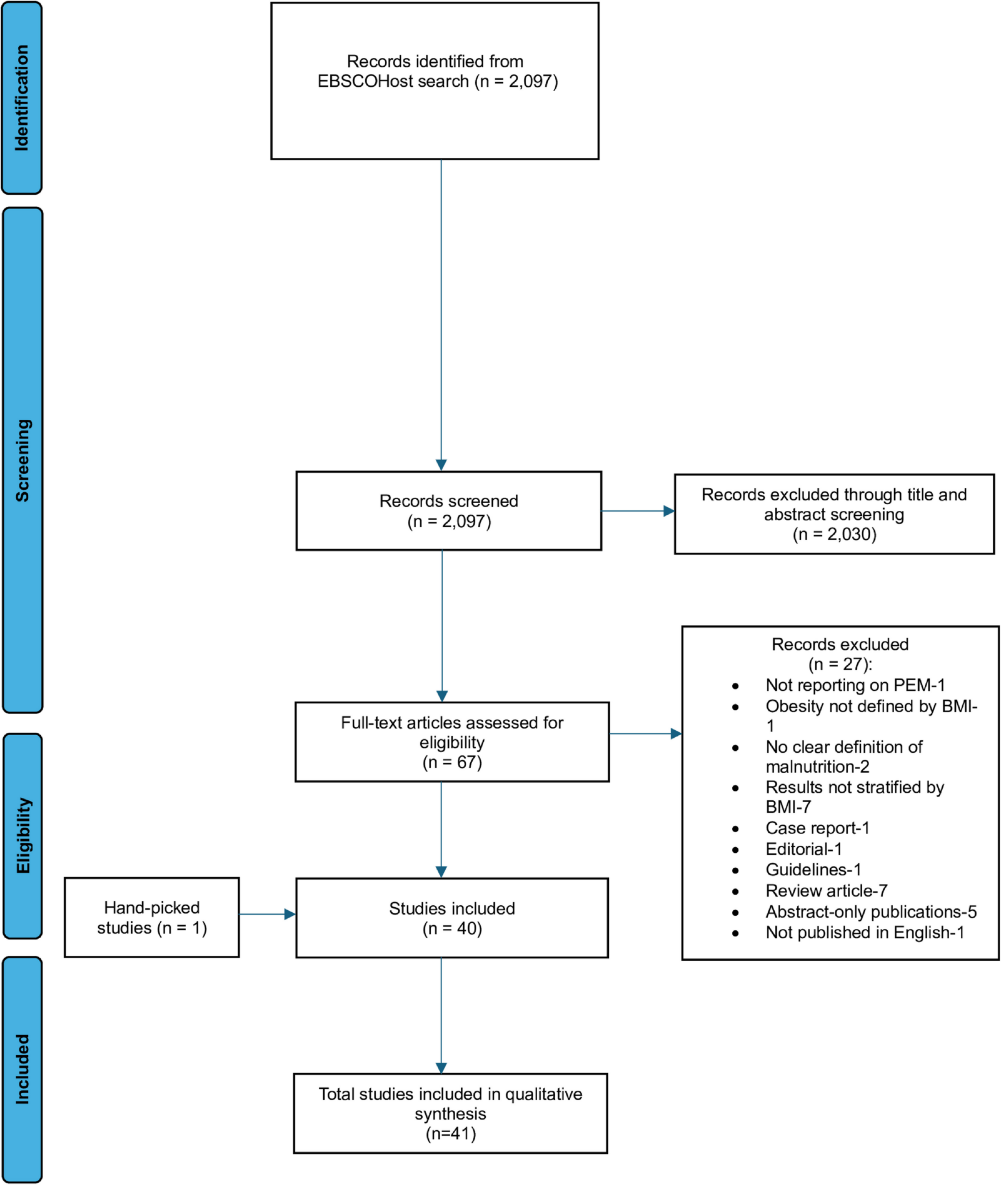
PRISMA‐ScR flowchart diagram for search findings and study selection on malnutrition screening and diagnosis in individuals with obesity.

The search identified a total of 2097 records from the database search. Following a comprehensive screening process, 67 full‐text articles were assessed for eligibility based on predefined criteria; of these, 41 met the inclusion criteria and were included in this scoping review. Figure 1 presents a summary of the evidence selection process, adhering to the PRISMA‐ScR guidelines.

### 4.2 Study Characteristics

Table 2 summarizes the selected studies, collectively involving 420,498 participants. These studies were conducted across developed and developing countries, spanning four continents. While predominantly set in hospitals, the study settings extended to outpatient facilities, community settings, and nursing homes, offering a comprehensive exploration of malnutrition across various contexts. The participant population included both inpatients and outpatients, with diseases ranging from acute conditions such as hip fractures and acute myocardial infarction to chronic illnesses such as cancer and heart failure.

**TABLE 2:** Overview of screening and diagnostic methods for malnutrition in individuals with obesity.

Author, publication year	Country	Population sample size (*n*)	Setting	Age (years)	Screening method/tool	Diagnosis of malnutrition	Relevant remarks on the method/tool
Agarwal 2019 [35]	Australia and New Zealand	Acute care patients (3122)	Hospital	≥ 18	‐MST‐SGA	‐ MST score ≥ 2 ‐ ICD‐10‐AM definition of BMI < 18.5 kg/m^2^ or unintentional loss of weight > 5% with evidence of suboptimal intake resulting in subcutaneous fat and/or muscle wasting	N/A
Arora 2018 [36]	USA	Radical cystectomy patients (2055)	Hospital	IQR 69 (62–67)	‐ Preoperative albumin level ‐Preoperative weight loss	‐ Hypoalbuminemia < 3.5 g/dL ‐ Significant (> 10%) weight loss	N/A
Bell 2021 [37]	Australia	Hip fracture inpatients (127)	Hospital	48–97	‐ICD10‐AM (PEM) criteria	ICD‐10‐AM definition of BMI < 18.5 kg/m^2^ or unintentional loss of weight > 5% with evidence of suboptimal intake resulting in subcutaneous fat and/or muscle wasting	“the ICD10‐AM protein‐energy malnutrition codes include criteria in addition to the BMI in an attempt to identify such patients with healthy, overweight or obese malnutrition. These criteria have recently been demonstrated as having the strongest concurrent and predictive validity in acute hip fracture inpatients”
Burman 2022 [38]	Sweden	Nursing home residents (47,686)	Nursing home	≥65	MNA‐SF	**a**	N/A
Buzney 2022 [39]	USA	Total shoulder arthroplasty patients (12,881)	Hospital	69.0 ± 9.6	Serum albumin level	Hypoalbuminemia < 3.5 mg/dL	N/A
Billeter 2015 [40]	Germany	Bariatric surgery patients with T2DM (20)	Hospital	18–70	Serum concentrations of albumin	Hypoalbuminemia < 35 g/L	N/A
Chalopin 2023 [41]	France	Postbariatric surgery patients (18)	Hospital	42.2 ± 10.4	‐Serum albumin and prealbumin concentrations	GLIM	N/A
Chiang 2023 [42]	USA	Breast reconstruction surgery patients (10,865)	Hospital	IQR 45–60	Preoperative albumin level	Hypoalbuminemia < 3.5 g/dL	N/A
Chien 2021 [43]	Taiwan	Asymptomatic general population (5300)	Hospital	49.6 ± 11.4	‐Serum albumin concentration ‐ PNI	‐Serum albumin concentration < 45 g/L ‐PNI < 55 ‐GLIM	N/A
Chen 2024 [44]	China	Acute coronary syndrome patients (21,651)	Hospital	64.8 ± 11.3	PNI	PNI < 38	“However, previous studies that have examined the “obesity paradox” have often failed to account for the influence of malnutrition, which may be an indicator of patients' risk stratification and a key factor in understanding the paradox in patients with coronary artery disease. To address this, nutritional information can provide valuable insights, and the PNI has emerged as a widely used tool for evaluating nutritional status and disease outcomes.”
Chen 2024 [45]	China	Colorectal cancer patients (850)	Hospital	> 18	‐NRS 2002 ‐HGS	‐NRS 2002 score ≥ 3 ‐Low HGS defined as < 18 kg for women and < 26 kg for men ‐GLIM	“HGS is a simple and effective indicator for evaluating body function and metabolic status in overweight and obese patients and has been also associated with the increased risk of metabolic syndrome, diabetes, and other diseases. Emerging evidence suggests that the combination of the GLIM criteria and HGS may be a valuable tool for identifying subclinical malnutrition and predicting clinical outcomes in patients with cancer. However, few studies have explored its application in overweight/obese populations, which is a critical but understudied area of research. Evaluating the potential of this novel approach in managing overweight CRC patients is necessary to optimize treatment and prognosis.”
Courtney 2015 [46]	USA	Primary joint arthroplasty patients (670)	Hospital	60.86 ± 1.86	Serum albumin level	Serum albumin level < 3.5 mg/dL	N/A
de Oliviera 2023 [47]	Brazil	Overweight patients (643)	Hospital	≥ 20	‐NRS 2002 ‐MNA‐SF	**a**	N/A
Donini 2014 [34]	Italy	Underweight and overweight patients (396)	Hospital	60.6 ± 17.0 (men) 62.6 ± 18.0 (women)	JaNuS	**a**	The precision and reliability of any screening tool assessing risk of undernutrition, when applied to obese patients, are likely to show these patients as having an acceptable nutritional status. Therefore, obese subjects, by definition, do not seem to be at nutritional risk and clinical conditions, such as concomitant sarcopenia, unintentional weight loss, and reduced food intake, are not correctly identified by the current screening tests. Since screening tools aimed at identifying the copresence of over and undernutrition are scarce, we aimed to develop and validate a screening tool for the easy detection and reporting of both undernutrition and overnutrition, specifically identifying the clinical conditions when the two types of malnutrition coexist.
Elliot 2023 [48]	Australia	Hospitalized patients (513)	Hospital	≥ 18	‐MST	‐ICD‐10‐AM definition of BMI < 18.5 kg/m2 or unintentional loss of weight > 5% with evidence of suboptimal intake resulting in subcutaneous fat and/or muscle wasting	N/A
Erb 2014 [49]	USA	Hemodialysis patients (253)	Hospital	63.5 ± 14.3	‐7‐Point SGA (Detsky A/B/C conversion) ‐Serum albumin concentrations	‐SGA < 5	“In CKD patients receiving maintenance hemodialysis (HD), a validated way to assess the nutritional status is through the SGA. The physical examination, widely regarded as the most important part of the SGA, assesses muscle and fat wasting at key locations on the body. Although a large amount of data have shown the validity of the SGA in HD patients, recent studies question its validity among obese patients because SGA only assesses the undernourished side of malnutrition. Given the prevalence of obesity in this population, understanding the validity of the SGA in obesity is critical; however, no data are available on the subject. Serum albumin, although not a pure nutrition marker, is a strong predictor of mortality in the HD population; thus, its relationship to the SGA in obese patients is one of interest.”
Ernst 2024 [50]	13 European countries*	Nursing home residents (11,327)	Nursing home	≥ 65	‐Malnutrition identification according to staff (yes, at risk, no) ‐MNA‐SF	**a**	N/A
Fieber 2018 [51]	USA	Bariatric surgery patients (106,577)	Hospital	≥ 18	Preoperative albumin level	Hypoalbuminemia defined as < 3.5 g/dL	N/A
Fu 2016 [52]	USA	Total hip arthroplasty patients (20,210)	Hospital	≥ 18	Preoperative albumin level	Hypoalbuminemia defined as < 3.5 g/dL	“It has been proposed that although overweight and obese patients may not be calorie deficient, they may often be micronutrient and protein deficient. It is well described that preoperative malnutrition has been identified as an important risk factor for postoperative complications, with clinical significance as a potentially modifiable comorbidity.”
Fu 2017 [53]	USA	Total knee arthroplasty patients (71,599)	Hospital	≥ 18	Serum albumin level	Hypoalbuminemia defined as < 3.5 g/dL	N/A
Gioulbasinis 2014 [54]	France and Greece	Cancer patients (1469)	Hospital	French > 70; Greek ≥ 18	MNA	**a**	“By this means, MNA not only estimates energy stores—found in abundance in overweight/obese patients—but it additionally evaluates important aspects of functionality. Moreover, it accounts for alterations in food consumption and changes in body image. Completion of the protocol by any healthcare provider regularly will take < 100.”
Hertroijs 2012 [55]	The Netherlands	Rehabilitation patients (366)	Rehabilitation center	Mean age 55	‐Weight loss in last 1,3,6 months ‐BMI	Patients were defined as severely undernourished when they met one or more of the following criteria: BMI < 18.5 (or BMI < 20 for patients age ≥ 65 years) and/or > 5% unintentional weight loss in the past month and/or > 10% unintentional weight loss in the past 6 months. Patients age ≥ 65 years were defined as moderately undernourished if they met the following criteria: BMI 20–22 and/or 5%–10% unintentional weight loss in the past 6 months. Patients age < 65 years were defined as moderately undernourished with a BMI 18.5–20 and 5%–10% unintentional weight loss in the past 6 months	N/A
Huang 2024 [56]	China	Overweight and obese cancer patients (3499)	Hospital	64 (IQR:56–71)	‐NRS 2002 ‐PG‐SGA ‐CPI	PG‐SGA score ≥ 4 CPI < 0.65% for men and < 0.57% for women	“However, the prognostic performance of CC in overweight or obese patients may be limited because this population has a higher proportion of fat mass in clinical practice, the body surface area (BSA) is commonly used to calculate the doses of chemotherapeutic agents. Smith et al. proposed that BSA might be an effective indicator for differentiating adipose tissue from muscle tissue. This consideration arose from the fact that muscle tissue, being denser and heavier than fatty tissue, occupies significantly less physical space, and has an equivalent weight of 1 kg for both muscle and fat. Therefore, the present study aimed to define a novel CPI as the proportion of the cross‐sectional area of the CC to the BSA and comprehensively investigate its prognostic significance in overweight or obese patients with cancer.”
Holbert 2023 [57]	USA	Total joint arthroplasty patients (79,984)	Hospital	64.07 ± 0.30	Preoperative albumin levels	Hypoalbuminemia defined as < 3.5 g/dL	N/A
Huang 2021 [58]	China	Surgical gastric cancer patients (587)	Hospital	65.65 ± 0.40	‐NRS 2002	GLIM	“Concerning the difficulty of the assessment of nutritional status in the overweight and obese patients, we speculated that muscle quality, strength and gait speed could support the diagnosis of malnutrition in these patients, in terms of improving the recognition of adverse clinical outcomes.”
Jensen 2006 [33]	USA	Older adult patients (1324)	Hospital	≥ 65	NHOQ	**a**	“Currently available nutrition risk screening questionnaires for older persons have specifically focused upon recognition of undernutrition, underweight, and frailty. These instruments therefore lack established validity for overweight/obese persons and have not been systematically tested in this regard. Reliable body weights and circumferences can be difficult to obtain for obese persons. Such individuals may also suffer sarcopenic obesity and deconditioning without evident weight loss. Fluid retention and increased fat mass may mask erosion of muscle mass. Poor quality diets can result in micronutrient deficiencies among obese persons that are not detected by simple food frequency intake queries and may not have manifest physical examination findings. Our research team has therefore systematically developed a self‐report 14‐item NHOQ intended to identify overweight/obese persons at risk for functional decline and healthcare resource use.”
Kong 2023 [59]	Singapore	Acute myocardial infarction patients (1829)	Hospital	≥ 21	‐CONUT	**a**	N/A
Leibovitz 2013 [60]	Israel	Newly admitted patients (431)	Hospital	≥ 18	NRS 2002	**a**	“As low BMI is only one of the four NRS 2002 criteria for estimating malnutrition risk, patients with normal or even elevated BMI can still be at increased risk for malnutrition. Thus, overweight and obese hospitalized individuals might be expected to have reduced in‐hospital mortality risk; on the other hand, patients at increased risk for malnutrition are at increased risk for mortality.”
Major 2018 [61]	Poland	Bariatric surgery patients (553)	Hospital	18–65	‐Serum albumin level ‐Total lymphocyte count	‐Hypoalbuminemia defined as < 3.5 g/dL ‐ Total lymphocyte count < 1500/μL	“Preoperative screening with serum albumin, particularly in morbidly obese patients, can identify risk patients for complications following other than bariatric surgeries.”
Martin 2020 [62]	Canada	Head and neck cancer patients (1157)	Hospital	≥ 18	PG‐SGA SF	**a**	“… .recently demonstrated that CT‐defined sarcopenia and myosteatosis were prevalent in advanced cancer patients across all BMI classes, and these features were not captured by different levels of nutrition risk assigned by the MUST, MST, or the NRI. While these tools were not specifically developed to detect patients with sarcopenia or myosteatosis, it is of interest to clinical practice to understand and create awareness about the extent to which these occult features, associated with poor clinical outcomes, exist in patients with different degrees of nutrition risk. We aimed to evaluate nutrition risk in overweight and obese cancer patients using the PG‐SGA, which is a validated nutrition assessment tool endorsed by the Oncology Nutrition Dietetic Practice Group of the Academy of Nutrition & Dietetics, and it includes a short form (PG‐SGA SF) for nutrition risk screening.”
Özkaya 2019 [63]	Turkey	Overweight and obese elderly (187)	Nursing home	> 65	MNA‐SF	**a**	N/A
Ritz 2009 [64]	France	Gastric bypass surgery patients (110)	Hospital	42 ± 10	‐Weight loss ‐Plasma proteins levels (albumin and transthyretin)	Malnutrition was considered according to a consensus by the French Healthcare High Authority (Haute Autorité de Santé) criteria. Mild PEM was considered for a weight loss greater than 10%, an albumin concentration lower than 30 g/L, or a transthyretin concentration lower than 110 mg/L. Severe PEM was considered for a weight loss greater than 15%, an albumin concentration lower than 20 g/L, or a transthyretin concentration lower than 50 mg/L.	N/A
Robinson 2015 [65]	USA	ICU patients (6518)	Hospital	63.8 ± 16.5	Standardized evaluation by registered dietitian	To meet criteria for PEM, patients must have a combination of disease‐related weight loss, decrease in ideal body weight, overt muscle wasting, peripheral edema, inadequate kcal or protein intake, decrease in albumin, transferrin, or total lymphocyte count	N/A
Sousa‐Santos 2020 [66]	Portugal	Community‐dwelling older adults (1454)	Home/retirement home	≥ 65	MNA‐SF	**a**	N/A
Soysal 2022 [67]	Turkey	Older adult obese patients (1911)	Hospital	77.34 ± 8.0	MNA	**a**	“Therefore, current guidelines recommend routine screening of older adults for malnutrition and risk of malnutrition, even if they are overweight or obese. Indeed, recommending weight loss without screening for malnutrition to reduce orthopedic problems, cardiovascular and metabolic risk in geriatric obese may predispose the patient to the occurrence of harmful effects such as sarcopenic obesity associated with weight loss. Therefore, the frequency of malnutrition and malnutrition risk in the geriatric and obese as well as the factors causing nutritional deterioration in this population need to be identified. However, to the best of the authors' knowledge, there is no study on this so far. In only one study conducted in a geriatric outpatient clinic, but using the MNA‐SF form, it was found that 33.7% of older patients were diagnosed with both obesity and malnutrition or were at risk of malnutrition. However, associated factors were not investigated in this study. Thus, the aim of the present study is to investigate the prevalence of undernutrition (i.e., malnutrition and malnutrition risk) and related factors in older obese patients.”
Sulmont‐Rossé 2022 [68]	France	Community‐dwelling older adults (782)	Residential, Nursing home	≥65	MNA	**a**	N/A
Uemura 2020 [69]	Japan	Acute heart failure inpatients (170)	Hospital	57–79	CONUT	**a**	“The prevalence of malnutrition was relatively discordant among screening tools especially for the mild malnutrition in patients with overweight/obesity, and the prognostic impact of nutritional status in patients with acute HF with an elevated BMI has not been fully elucidated.”
Van der Louw 2020 [70]	USA	ICU patients with acute myeloid leukemia (145)	Hospital	> 18	‐BMI, weight loss over 3 months before hospital admission ‐Modified NUTRIC score ‐Plasma albumin level	Malnutrition defined > 2 of the following as documented by a registered dietician: insufficient energy intake, weight loss > 5% over 1 month or > 7.5% over 3 months, loss of muscle mass, loss of subcutaneous fat, fluid accumulation or reduced grip strength	N/A
van Vliet 2021 [71]	The Netherlands	Surgical and general ward patients (430)	Hospital	≥ 18	‐MUST ‐PG‐SGA SF	**a**	“… there is a need for suitable instruments to identify patients at high risk for disease‐related malnutrition, presenting with a different phenotype than low BMI or critical weight loss. Such an alternative is offered by the PG‐SGASF. This tool can be considered as an alternative screening tool for clinical practice, as it includes items on weight history irrespective of BMI, food intake, nutrition impact symptoms (e.g., nausea, problems swallowing, diarrhea), and activity and functioning.”
Vidot 2019 [72]	Australia	Patients listed for liver transplantation (205)	Hospital	52 ± 0.7	Liver‐specific SGA	**a**	N/A
Zhou 2023 [73]	China	Rectal cancer surgical patients (624)	Hospital	≥ 18	‐NRS 2002	GLIM	“The GLIM criteria offer a major conceptual advancement in the diagnosis of malnutrition, even in patients with high BMI and adiposity. Different combinations of phenotypical and etiological criteria allowed for a wide range of GLIM applications.”

*Note:* **a**: The screening tool was used for diagnosis with the normal cutoffs/categories as designed and validated for the tool.

Abbreviations: BMI, body mass index; CONUT, controlling nutritional status; CPI, calf proportion index; GLIM, global leadership initiative on malnutrition; HGS, hand grip strength; ICD‐10‐AM, International Classification of Diseases, 10th Revision, Australian Modification; JaNUS, just a nutrition screening; MNA, mini nutritional assessment; MNA‐SF, mini nutritional assessment—short form; MST, malnutrition screening tool; MUST, malnutrition universal screening tool; N/A, not available; NHOQ, Nutrition Health Outcomes Questionnaire; NRS 2002, nutritional risk screening 2002; NUTRIC, nutrition risk in the critically ill; PEM, protein‐energy malnutrition; PG‐SGA, patient‐generated subjective global assessment; PG‐SGA‐SF, patient‐generated subjective global assessment—short form; PNI, prognostic nutritional index; SGA, subjective global assessment.

* Austria, Belgium, Czech Republic, Germany, Italy, Luxembourg, Norway, Poland, Portugal, Serbia and Montenegro, Slovenia, and Switzerland.

The assessment of malnutrition risk and/or nutritional status across various settings and populations was conducted using three main methods: (1) blood markers (mostly in hospital settings) (such as albumin, transthyretin, and lymphocyte count); (2) malnutrition screening tools (including MNA[‐SF], NRS 2002, MST, and CONUT); and (3) physical/etiologic assessments (weight loss, muscle mass/strength, and food intake). The GLIM diagnostic criteria were the most commonly used criteria for diagnosing malnutrition. Table 2 also provides insights into the validity and applicability of these methods for identifying malnutrition in populations with obesity. Despite their common use, none of these methods provided obesity‐specific cutoff values for screening and diagnosing malnutrition. While some cutoff values were provided for blood markers, they were generalized and not specifically tailored for populations with obesity.

Additionally, the search identified a manuscript by Jensen, detailing the development of a 14‐item screening tool, the Nutrition Health Outcomes Questionnaire (NHOQ), to assess malnutrition in older adults with obesity [33]. A handpicked article by Donini et al. introduced the Just a Nutritional Screening tool (JaNuS), specifically developed for (pre‐)geriatric populations to address both over‐ and undernutrition [34]. These articles were included as they closely aligned with the review's objectives.

## 5 Discussion

This scoping review represents the first comprehensive effort to explore the literature on identifying malnutrition risk and diagnosing malnutrition in individuals with obesity. The assessment of malnutrition risk and/or nutritional status in this population, as reported in the included studies, predominantly relies on three primary methods: blood markers (such as albumin, transthyretin, and lymphocyte count), malnutrition screening tools (including MNA[‐SF], NRS 2002, MST, and CONUT), and physical/etiologic assessments (weight loss, muscle mass/strength, and food intake). For a formal diagnosis, the GLIM criteria were most commonly applied. Notably, these methods closely resemble those used for populations with a normal BMI (18.5–24.9 kg/m^2^) [74], prompting a critical examination of their applicability and effectiveness within the unique context of obesity complicated by malnutrition.

In hospital settings, routine blood tests that assess indicators such as albumin, transthyretin, and lymphocyte count were once used as diagnostic markers for malnutrition [75]. However, recent studies have questioned their reliability due to limitations in sensitivity and specificity, particularly in the presence of inflammation [76, 77, 78]. In addition to inflammation caused by acute disease, conditions such as obesity can trigger low‐grade chronic inflammation, resulting in the production of proinflammatory cytokines [79, 80]. This alters the levels of these markers and compromises their accuracy in identifying malnutrition [77, 81]. Moreover, metabolic disturbances associated with obesity, such as nonalcoholic fatty liver disease (NAFLD), contribute to increased vascular permeability [82, 83]. These conditions can contribute to hypoalbuminemia, low transthyretin levels, and reduced lymphocyte counts [83, 84], further complicating the interpretation of these blood markers of malnutrition risk [85]. Hence, it is important to recognize that malnutrition is a complex condition that cannot be adequately assessed through blood markers alone [86]. This limitation applies not only to individuals with obesity but also to those with a normal BMI [86].

Various malnutrition screening tools, including the MNA, MNA‐SF, MUST, and NRS 2002, were utilized to identify malnutrition risk in the populations studied. However, their applicability to individuals with obesity remains uncertain due to insufficient evidence validating their use in this population. A screening tool must undergo validation to ensure its accuracy in measuring specific parameters within a particular population [87, 88], considering factors such as age, disease, and ethnicity when establishing cutoff values [89, 90]. Failure to consider these factors may yield inaccurate outcomes when using these tools in nontarget populations. Essentially, a positive screening result does not equate to a diagnosis of malnutrition, especially with a nonvalidated tool, as screening tools are not diagnostic [91]. For example, while the NRS 2002 is validated for identifying malnutrition risk in hospital settings, and the MNA is validated for use in geriatric populations in nursing homes or community settings [92], neither has been validated for use in populations with obesity. Nevertheless, despite their lack of validation in this population, these tools, along with others, were utilized for malnutrition screening in the individuals with obesity included in this scoping review.

Two studies in this review [59, 69] used the CONUT screening tool, which assesses nutritional status using serum albumin, total lymphocyte count, and cholesterol levels [93]. However, this tool may not be ideal as a malnutrition screening tool due to its lack of consideration of body composition and reliance on blood markers. The challenge of distinguishing whether abnormal blood marker levels are caused by malnutrition, underlying disease, or disease‐associated inflammation further complicates the use of the CONUT as a malnutrition screening tool [91]. This uncertainty raises concerns about its specificity. Moreover, using this tool for screening individuals with obesity could result in an overestimation of malnutrition risk and misidentification of at‐risk individuals.

Although most screening methods lacked validation in populations with obesity, two studies utilized tools specifically developed for and validated in such populations: the NHOQ and JaNuS tool [33, 34]. The NHOQ outlines the development of a 14‐item screening tool for malnutrition in older adults with obesity [33]. While it lacks formal validation and a clearly defined scoring methodology, the determinants described in the tool may inform the development of a universal screening tool tailored to obesity. Reliability testing indicated that the questionnaire addressed five key domains: cardiovascular disease, general health, functional status, diet quality, and weight reduction [33]. The other tool, the JaNuS, screens for both over‐ and undernutrition separately, enabling a patient to score positively for one or both conditions [34]. However, the tool's reliance on blood markers of low albumin and low lymphocyte count to indicate nutritional status limits its reliability when applied to individuals with obesity [84, 86]. In addition, the screening component related to overnutrition in the JaNuS tool focuses on obesity‐related conditions such as metabolic syndrome [34]. Nonetheless, other components of the tool may offer valuable insights for developing an ideal screening tool for malnutrition risk in individuals with obesity.

When screening individuals with obesity using current screening tools, questions related to BMI and recent unintentional weight loss are often not applicable. These questions typically account for 50% of the total score in many tools' scoring systems [94], but the high BMI associated with obesity can obscure clinically significant weight loss [21]. As a result, the cutoff points in these tools, originally designed for individuals with normal or low BMI, may not be valid for those with obesity, potentially leading to misclassification and missed diagnoses of malnutrition. Consequently, the focus of these tools shifts towards other factors such as mobility, psychological stress, disease severity, and dietary intake, resembling geriatric screening tools [91]. For example, in the MNA‐SF [95], if an individual with obesity (characterized by a high BMI and calf/arm circumference) has not experienced unintentional weight loss in the last 3 months, the screening process would primarily rely on factors like dietary intake, mobility, and (neuro)psychological stress, highlighting aspects often associated with ageing [96]. As a result, a positive screening may indicate a risk unrelated to malnutrition. This emphasizes the need for screening tools to adapt and account for the unique characteristics and challenges present in individuals with obesity, beyond their BMI status and anthropometric measurements.

In some included studies, all of which were of Australian origin [35, 37, 48], the ICD‐10‐AM was used to classify malnutrition [97]. Originally designed as a disease coding system, the ICD‐10‐AM framework includes a range of codes specifically for malnutrition, providing classification of conditions such as Kwashiorkor and PEM. These codes encompass various degrees of severity, i.e., mild, moderate, or severe PEM, as well as malnutrition associated with diseases such as cancer and other chronic illnesses [97]. Malnutrition codes, ranging from E40 to E46, include Kwashiorkor (E40), Nutritional Marasmus (E41), and PEM, with distinctions for severity, such as E44.0 for moderate and E44.1 for mild PEM [97]. However, the ICD‐10‐AM may not fully cover the spectrum of malnutrition [97], especially in individuals with obesity. This coding system lacks defined cutoff values for commonly used nutritional assessment parameters such as BMI, mid‐upper arm circumference (MUAC), and laboratory markers, which are typically established by consensus guidelines from organizations such as the World Health Organization (WHO). Recognizing these limitations, efforts have been made to revise the classification system. A proposal has been submitted to the WHO to update the adult malnutrition classification by including a category for malnutrition that does not rely on the standard BMI cutoff of 18.5 kg/m^2^ [74]. Further specifications would address malnutrition both with and without factors such as hunger‐related malnutrition. A decision regarding these changes is anticipated to be made in 2025 [98].

Several studies [41, 43, 45, 58, 73] applied the GLIM criteria to diagnose malnutrition following screening [26]. These criteria represent an advancement over the 2002 guidelines of the European Society for Clinical Nutrition and Metabolism (ESPEN) [14], offering more options for diagnosing malnutrition beyond low BMI and recent unintentional weight loss [26]. However, the GLIM criteria have not been validated specifically for individuals with obesity and lack tailored cutoffs for this population [4]. Moreover, the application of the GLIM criteria in these studies was flawed as they neglected to consider all five criteria. Instead, they focused on selecting only one criterion from each category: phenotypic (unintentional weight loss, low BMI, and muscle mass) and etiologic criteria (reduced food intake and inflammation) for the diagnosis [15]. Given that all participants were patients, it was assumed that one etiologic criterion (disease burden) was already met. Consequently, the diagnosis of malnutrition was primarily based on the significant/nonvolitional weight loss and low muscle mass criteria [58, 73].

Applying the GLIM criteria to individuals with obesity presents challenges, particularly in identifying appropriate cutoff values for both phenotypic and etiologic criteria [4]. To improve applicability in this population, adaptations to the GLIM criteria may be necessary. For instance, while techniques like bioelectrical impedance analysis (BIA), dual‐energy X‐ray absorptiometry (DEXA), and computed tomography (CT) are commonly used to assess muscle mass, DEXA and CT have limitations such as weight restrictions and reduced reliability in individuals with obesity [4]. As a practical alternative, anthropometric measurements such as calf circumference could be considered. However, standard cutoff points for calf circumference may not be appropriate for individuals with obesity, highlighting the need to consider age‐ and BMI‐specific cutoff points as previously developed [20].

Additionally, relying solely on a 50% reduction in food intake may not accurately indicateinadequate nutrient intake in individuals with obesity [99]. This limitation arises from challenges posed by higher nutritional requirements, varying eating patterns, and potential impacts of bariatric surgery [4]. While indirect calorimetry may offer a more precise method for evaluating energy expenditure and can enhance understanding of dietary needs within this population [100, 101], it is not a standard screening tool. Also, it may not be practical for widespread use [100]. Moreover, prediction equations for energy expenditure in individuals with obesity are often inadequate [102], further complicating the interpretation of dietary assessments based on this criterion.

Within the GLIM criteria, inflammation is considered a marker of disease burden rather than nutritional status and is evaluated alongside other criteria [103]. However, individuals with obesity often exhibit elevated C‐reactive protein (CRP) levels due to consistent low‐grade inflammation, potentially leading to false positives in the GLIM assessment [79, 80, 104]. The cutoff level in the GLIM is 3.0 mg/dL, and low‐grade inflammation is often already higher [103]. Hence, there is a need to re‐evaluate the inflammation criterion within GLIM and recognize the potential for false positives when assessing malnutrition in individuals with obesity. The well‐established link between obesity and increased CRP levels is attributed to the pathophysiological mechanism in which the liver processes excess free fatty acids, stimulating cytokine release from adipose tissue. These cytokines, particularly interleukin‐6 (IL‐6), stimulate CRP production [105]. Studies by Klisic et al. and Dayal et al. support this association, showing higher CRP levels in individuals who are overweight compared to those with a healthy weight [106, 107].

This scoping review has both strengths and limitations. It employed an iterative process, deviating from the structured methodology standard of systematic reviews. The search not only set the foundation for further study investigations but also uncovered unexpected findings. One notable aspect was the unconventional inclusion criterion of BMI stratification, which led to a non‐systematic search for studies that explicitly incorporated this factor. Our goal was to include studies assessing malnutrition rates in obesity, but most studies did not explicitly state this as their primary aim. While many studies report prevalence rates by risk group, including BMI categories [24], they do not specifically focus on assessing malnutrition in individuals with obesity. Nevertheless, since most studies that examined malnutrition risk across different BMI categories stratified their results accordingly, this method was adopted as part of the inclusion criteria. Incorporating this approach helped refine the scope and relevance of the review.

We limited our screening to a single database, EBSCOhost (Medline), consciously accepting the risk of missing other relevant evidence. This approach aligns with the purpose of scoping reviews, which is to provide an overview of existing evidence rather than achieve completeness [108]. In our review, we identified three main methods for screening for malnutrition risk. However, very few papers reported on the validity and reliability of these methods for this population. The use of varying terminology and definitions of malnutrition across the literature made it challenging to capture all relevant information. However, this is unlikely to greatly affect the overall findings.

## 6 Conclusion

Screening for malnutrition risk and diagnosing malnutrition in individuals with obesity is a complex challenge that requires a re‐evaluation and adjustment of existing methods. This scoping review highlights the limitations of current screening and diagnosis methods, which are often based on criteria designed for individuals with a healthy weight. Despite their widespread use, these methods lack the reliability and validity to assess malnutrition in individuals with obesity. Blood markers can exaggerate the risk of malnutrition due to their sensitivity to factors such as inflammation and hydration status, while primary phenotypical markers, including BMI and recent unintentional weight loss, may result in an underdiagnosis of malnutrition.

To improve outcomes, it is essential to adapt these methods by incorporating specific cutoff values for weight loss, muscle mass, and other obesity‐related factors. Such changes will equip healthcare professionals to identify malnutrition risks earlier, facilitating timely interventions and ultimately improving patient care.

## Conflicts of Interest

The authors declare no conflicts of interest.

## Data Availability

The data that support the findings of this study are available from the corresponding author upon reasonable request.
